# Depletion of giant ANK2 in monkeys causes drastic brain volume loss

**DOI:** 10.1038/s41421-021-00336-4

**Published:** 2021-11-30

**Authors:** Dong-Dong Qin, Jian-Kui Zhou, Xie-Chao He, Xiang-Yu Shen, Cong Li, Huan-Zhi Chen, Lan-Zhen Yan, Zheng-Fei Hu, Xiang Li, Long-Bao Lv, Yong-Gang Yao, Zheng Wang, Xing-Xu Huang, Xin-Tian Hu, Ping Zheng

**Affiliations:** 1 Key Laboratory of Animal Models and Human Disease Mechanisms, Chinese Academy of Sciences and Yunnan Province, Kunming Institute of Zoology, Chinese Academy of Sciences, Kunming, Yunnan China; 2 School of Basic Medical Sciences, Yunnan University of Chinese Medicine, Kunming, Yunnan China; 3 Precise Genome Engineering Center, School of Life Sciences, Guangzhou University, Guangzhou, Guangdong China; 4 Primate Facility, National Research Facility for Phenotypic & Genetic Analysis of Model Animals, and National Resource Center for Non-Human Primates, Kunming Institute of Zoology, Chinese Academy of Sciences, Kunming, Yunnan China; 5 Institute of Neuroscience, State Key Laboratory of Neuroscience, Chinese Academy of Sciences, Shanghai, China; 6 State Key Laboratory of Genetic Resources and Evolution, Kunming Institute of Zoology, Chinese Academy of Sciences, Kunming, Yunnan China; 7 Kunming College of Life Science, University of Chinese Academy of Sciences, Kunming, Yunnan China; 8 CAS Center for Excellence in Brain Science and Intelligence Technology, Chinese Academy of Sciences, Shanghai, China; 9 KIZ/CUHK Joint Laboratory of Bioresources and Molecular Research in Common Diseases, Kunming Institute of Zoology, Chinese Academy of Sciences, Kunming, Yunnan China; 10 School of Life Science and Technology, Shanghai Tech University, Shanghai, China; 11 Yunnan Key Laboratory of Animal Reproduction, Kunming Institute of Zoology, Chinese Academy of Sciences, Kunming, China; 12 Center for Excellence in Animal Evolution and Genetics, Chinese Academy of Sciences, Kunming, Yunnan China

**Keywords:** Cell biology, Developmental biology

Dear Editor,

Autism spectrum disorders (ASDs) are heritable neurodevelopmental disabilities with core symptoms of impaired reciprocal social behaviors and restrictive or repetitive behaviors^1^. ASDs are relatively common developmental neuropsychiatric disorders and important public health issues, affecting around 1%–2% of the population^2^. ASDs have been classified into syndromic and nonsyndromic (also called classic or idiopathic). Most syndromic ASDs have a genetic basis and around 1000 candidate risk genes have been identified according to the Simons Foundation Autism Research Initiative (SFARI Gene: https://www.sfari.org/resource/sfari-gene/). Unlike syndromic ASDs, the etiologies of most nonsyndromic ASD cases are largely unknown and very limited genes have been implicated in that.

*ANK2* is a member of ankryin gene family and is transcribed into two major isoforms via alternative splicing. The two isoforms produce 220 and 440 kDa polypeptides, termed ANK2 and giant ANK2, respectively^3^. Compared to ANK2, giant ANK2 has an additional fragment encoded by exon 37 (2066 amino acid residues in cynomolgus and rhesus monkeys, and 2085 amino acid residues in human). Unlike ANK2 which displays broad expression in many tissues including nervous system, giant ANK2 is restrictively expressed in nervous system^4^. Several giant ANK2-specific mutations (p.P1843S, p.R2608 frameshift, and p.E3429V), which locate in exon 37, were identified in nonsyndromic ASD patients^4^. In addition, a recent study of giant ANK2 deletion in mice revealed that loss of giant ANK2 has no effects on brain structure, but displays mild impairment on selected communicative and social behaviors^4^. These studies suggested that giant ANK2 might be a potential genetic factor involved in nonsyndromic ASDs. Although the laboratory mice are widely employed to decipher the molecular and cellular mechanisms underlying ASDs, rodent models have the limitation that the animals are phylogenetically distant from human. Non-human primates share higher degree of similarity with humans in genome sequence and physiology^5^. Specifically, monkeys have a well-developed prefrontal cortex and display a repertoire of behaviors that are more relevant to ASDs^5^. Several recent studies have established reliable syndromic ASDs monkey models by overexpression or knockout of *MECP2* or *SHANK3* in cynomolgus monkeys^6–8^.

In this study, we evaluated the function of giant ANK2 and its relevance to human nonsyndromic ASDs by using CRISPR/Cas9 gene-edited cynomolgus monkeys (*Macaca fascicularis*) and rhesus monkeys (*Macaca mulatta*) in which giant ANK2 was specifically knocked out whilst ANK2 remained intact. Two sgRNAs were designed to target distinct sites at exon 37 encoding unique fragment of giant ANK2 (Fig. 1a). Evaluating the cynomolgus monkey pre-implantation embryos revealed high editing efficiency (Supplementary Fig. S1). After embryo transfer, we obtained three pregnancies in cynomolgus monkeys and six in rhesus monkeys, respectively. All fetuses developed to full term. Unfortunately, one cynomolgus monkey and three rhesus monkeys died within 17 days after birth. Genotyping of the nine monkeys was conducted using genomic DNA extracted from the umbilical cord, ear skin, or blood samples (Supplementary Table S1). Among the five live monkeys, two cynomolgus monkeys and two rhesus monkeys contained frame-shift mutations at both alleles (Supplementary Fig. S2 and Table S1). These frame-shift mutations caused premature translational stop and possibly a complete loss of the functional giant ANK2. Notably, one rhesus monkey (T113) harbored only a small fragment deletion and missense mutations (Supplementary Fig. S2a), which may not significantly alter giant ANK2 functions. We collected eight different tissues from dead monkeys and confirmed the mutations by using Sanger sequencing (Supplementary Fig. S3). We also validated the mutations of *ANK2* at both alleles in brain or liver tissue from three dead monkeys and in peripheral blood samples from all five live monkeys with successful genetic modification using the second-generation sequencing technologies (Supplementary Figs. S2b, S3, and S4). Furthermore, we examined the protein expression of ANK2 and giant ANK2 in the brain of one dead monkey (T114, frameshift). Due to the extremely large size of giant ANK2 (~440 kDa), we had difficulty in detecting this isoform in wild-type (WT) control. However, we confirmed that the ANK2 isoform (~220 kDa) was not affected in knockout (KO) monkey (Supplementary Fig. S2c), demonstrating the specific mutation of giant ANK2 isoform.

**Fig. 1:**
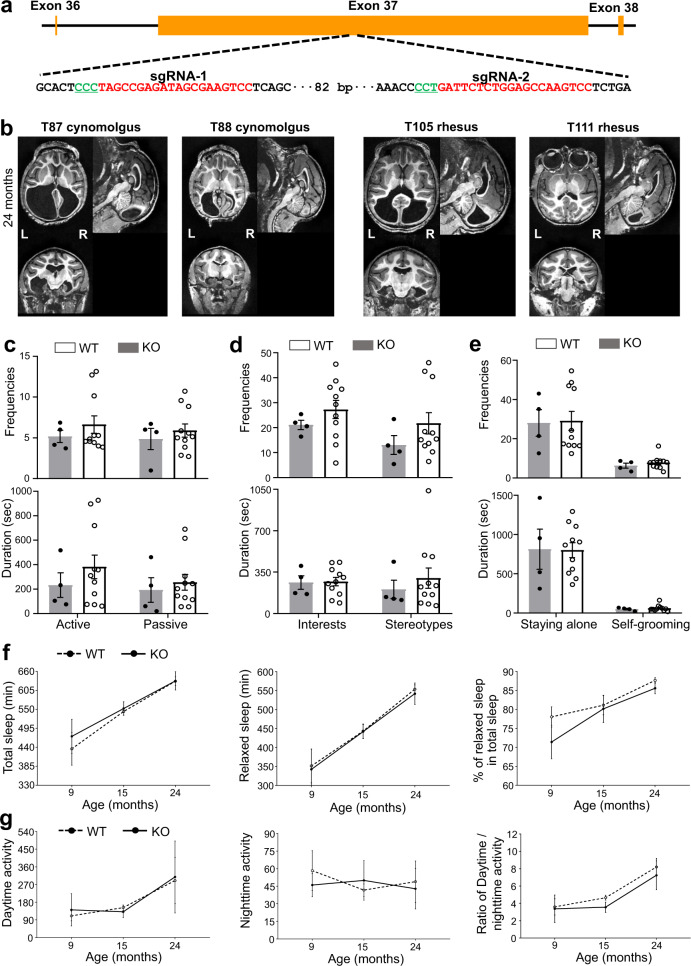
Giant ANK2 knockout monkeys display reproducible brain volume loss, but do not show ASDs symptoms and have normal sleep–wake cycle. **a** Schematic diagram showing two sgRNAs which target the specific exon of giant ANK2. **b** Example axial, sagittal, and coronal slices of structural images of each knockout (KO) monkey at 24-month. L-left; R-right. **c–e** Frequencies and durations of both active and passive social interaction (**c**), exploratory and stereotypical behaviors (**d**), staying alone and self-grooming (**e**) between KO and WT monkeys. **f**, **g** No significant difference of the sleep state (**f**) (including the duration of total sleep and relaxed sleep, and the percentage of relaxed sleep in total sleep) and the activity (**g**) (including day-time activity, night-time activity and ratio of day-time/night-time activity) between KO and WT monkeys. All data in (**c–g**) were presented as means ± SEM.

We firstly examined the possible influence of giant ANK2 mutation on brain structures. WT monkeys with matched ages and growth conditions were used as controls and were raised together with the mutant monkeys (Supplementary Table S1). Magnetic resonance imaging (MRI) scanning of monkey brains was performed using a United Imaging UMR 790 3T scanner (Shanghai, China) when monkeys were 6-month, 12-month, and 24-month old. Notably, a drastic brain structure change predicting the enlargement of lateral ventricles was reproducibly detected in two cynomolgus monkeys (T87 and T88) and two rhesus monkeys (T105 and T111), all of which carried frame-shift mutations and premature translational stop of giant ANK2. Moreover, the structural alternations were persistent at all examined ages. Some MRI scanning results were shown for the 24-month (Fig. 1b) and other ages as well (Supplementary Fig. S5a, b). Notably, the mutant rhesus monkey (T113) carrying only small fragment deletion and missense mutations did not display detectable brain structural abnormality compared to WT control (Supplementary Fig. S5c). This could be attributed to the mild change of protein sequence which may not alter protein functions. We therefore exclude this monkey (T113) from the following analyses.

Next, we conducted a quantitative analysis on structural MRI data that were collected at 24-month to evaluate the brain volumetric alterations. Each monkey’s brain was registered and segmented into 94 sub-regions based on the brain atlases of rhesus macaque (F99) and cynomolgus macaque (Cyno162)^9,10^. The structural MRI data for each mutant monkey was compared with specie-specific standard brain atlas instead of species-matched WT controls in this study, as these standard brain atlases were constructed by using more WT individuals^9,10^. The loss of gray matter volume (GMV) in each brain region was defined as the percentage of missing GMV (dark signal in T1-weighted MRI images) compared to the specie-specific standard brain atlas, i.e., dividing the missing GMV volume by the standardized regional GMV of the brain atlases^9,10^. The total brain volume of individuals was normalized by the total volume of the atlas. The average percentages of regional GMV loss for each brain region throughout the whole brain were shown (Supplementary Fig. S6a). The distribution of regional GMV loss in individual mutant monkeys was plotted as a violin plot (Supplementary Fig. S6b), and the top 20 brain regions with the largest GMV loss in mutant monkeys compared to WT controls were listed (Supplementary Fig. S6c). Regions that exhibited consistent and marked volumetric alteration in mutant monkeys were predominantly located in left visual area 1, left visual area 2, left anterior visual area, left ventral temporal cortex, and right medial frontal cortex.

To detect whether giant ANK2 depletion causes the core symptoms of ASDs, we performed a serial of behavioral tests including social interaction, environmental exploration, stereotypical behaviors, staying alone, and self-grooming^6^. Compared to the age- and gender-matched WT control monkeys, the mutant monkeys did not show typical ASDs-like behaviors. The frequencies and durations of both active and passive social interaction were comparable between WT and mutant monkeys (Fig. 1c). Consistently, no obvious differences in exploration interests and stereotypes (Fig. 1d), as well as staying alone and self-grooming (Fig. 1e) were detected between mutant monkeys and WT controls. These data collectively suggest that giant ANK2 depletion in non-human primate does not cause ASDs-like behaviors. This is in sharp contrast to the observations in the mutant mice^4^.

Sleep is closely associated with neuroplasticity, brain development, and health. We wondered whether the brain defects in mutant monkeys could cause parahypnosis. The sleep–wake cycle was monitored at 9-month, 15-month, and 24-month by actigraphy, which is reliable to score the sleep state in monkeys^11^. The results showed that mutant monkeys at each age had normal total sleep and relaxed sleep when compared to their WT counterparts (*P*-values > 0.05). Consistently, the proportion of relaxed sleep in total sleep did not differ between mutant and WT monkeys (Fig. 1f). We also evaluated the day-time activity, night-time activity, and the ratio of the day-time/night-time activity. Mutant monkeys did not display any difference from WT in these respects either (Fig. 1g). In order to explore whether the age affected sleep–wake cycles between the two groups, the data were further analyzed in separate 2 (groups: KO vs WT) × 3 (age: 9-month, 15-month, and 24-month) repeated-measure ANOVAs, with age being the repeated-measure. No significant differences were observed in both sleep and activity patterns between the two groups (all *P*-values > 0.05).

In summary, specific depletion of giant ANK2 protein in monkeys did not induce nonsyndromic ASDs-like behaviors or sleep and activity pattern alterations. This finding does not support the loss of giant ANK2 as an ASDs factor. Unexpectedly, giant ANK2 depletion caused drastic brain structural alteration in all mutant monkeys. Thus, the functions of giant ANK2 are evolutionarily divergent between rodents and primates. In addition, four mutant monkeys with drastic loss of brain volume displayed normal basic brain functions. This was consistent with several previous reports on human cases. For instance, patients who underwent surgical removal of one hemisphere in childhood had normal intra-hemispheric connectivity, social responsiveness, full-scale intelligence quotient, psychomotor function, and executive control^12^. Two persons having severe hydrocephalus and drastic brain volume loss also showed normal brain functions^13,14^. On the other hand, our monkey models can be used to study the functional re-organization and plasticity of brains, for which the molecular mechanisms still remain elusive.

## Supplementary information

Supplementary information.
