# Supplementary material for: Depletion of giant ANK2 in monkeys causes drastic brain volume loss
Source: Cell Discov. 2021 Nov 30;7:113. doi: 10.1038/s41421-021-00336-4 (PMC8629971; doi:10.1038/s41421-021-00336-4)
Supplement: Supplementary file 1 — Supplementary information. [file 41421_2021_336_MOESM1_ESM.docx]

**Online Supplementary Materials**

**Materials and Methods**

**Animals**

The cynomolgus monkey (*Macaca fascicularis*) and rhesus monkey (*Macaca mulatta*) facility in this study is accredited by AAALAC International. All experimental protocols were approved by the Institutional Animal Care and Use Committee (IACUC) of Kunming Institute of Zoology, Chinese Academy of Sciences. Throughout the experiment, the monkeys were housed in a controlled environment (temperature: 22 ± 1^o^C, relative humidity: 50% ± 5%) with 12 hours light / 12 hours dark cycle (lights on at 07:00 a.m.). All animals were given commercial monkey diet twice a day with tap water ad libitum and were fed fruits and vegetables once daily. Routine veterinary care was provided by professional keepers and veterinarians during the entire process of experiments to ensure good health.

**CRISPR/Cas9-mediated *ANK2* targeted editing in monkeys and genotyping**

Superovulation, oocytes collection, intracytoplasmic sperm injection (ICSI), injection of CRISPR-Cas9 system, embryo transfer and pregnancy diagnosis were performed as previously described ^1^. Briefly, healthy female cynomolgus monkeys, ranging in age from 5 to 8 years old with regular menstrual cycles, were selected as oocyte donors for superovulation. The selected monkeys were intramuscularly injected with rhFSH (Recombinant Human FSH, Gonal F, Laboratories Serono) at the same time each day for 8 days and on the ninth day they were injected with rhCG (Recombinant Human Chorionic Gonadotropin alpha for Injection, Merck Serono). 32-35 hours after administration of rhCG, laparoscopy was used for oocyte collection. The collected oocytes were cultured in the pre-equilibrated maturation medium, and the metaphase II arrested oocytes were used to perform ICSI. The fertilization was confirmed for the presence of two pronuclei. The fertilized oocytes were injected with Cas9 mRNA (20 ng/μL) and sgRNAs (10 ng/μL or 20 ng/μL for each sgRNA). Injected embryos were cultured in chemically defined protein-free hamster embryo culture medium-9 (HECM-9, Millipore) to allow embryo development. Cleaved embryos of high quality at the two-cell to blastocyst stage in high sgRNA concentration group were transferred into the oviduct of the matched surrogate recipient monkeys. Typically, three embryos were transferred into each surrogate, and the pregnancy diagnosis was firstly performed by ultrasonography on 20–30 days after transfer.

The tissues of aborted fetuses, including placenta, umbilical cord, brain, heart, kidney, liver, lung, muscle, skin and/or ovary, were collected and then quick frozen in liquid nitrogen. For survivors, the placenta, umbilical cord, ear skin fibroblasts and/or peripheral blood were collected. The collected tissues were digested in lysis buffer (10 mM Tris-HCl, 0.4 M NaCl, 2 mM EDTA, 1% SDS and 100 mg/ml Proteinase K). The genomic DNA was extracted from lysate by phenol-chloroform, and the *ANK2* mutations were identified by T7E1 cleavage assay with T7 Endonuclease (NEB) and Sanger sequencing. The primer pairs for amplifying sgRNA-targeted regions of the *ANK2* gene were listed in Supplementary Table S2.

**Deep sequencing of the sgRNA targeted region of *ANK2* for validation**

To further validate and quantify the mutations introduced by Cas9/sgRNA, we performed a targeted sequencing of the sgRNA targeted region of *ANK2* by using the second-generation sequencing technologies (deep sequencing). We used genomic DNA isolated from peripheral blood samples of all five live monkeys and brain (T89 and T112) or liver (T114) tissues from dead monkeys with successful gene editing of *ANK2* as revealed by the TA-cloning sequencing. Briefly, targeted regions of *ANK2* were PCR amplified using primer pair ANK2-HTS-(1-17)-F and ANK2-HTS-R (Table S2) and were subjected to Illumina NovaSeq 6000 for high-throughput sequencing. Reads of individual sample from pool-sequencing were splitting by using Fastq-multx (V1.3.1) ^2^; paired-end reads mapping was conducted by BWA (V0.7.17) ^3^ and Samtools (V1.7) ^4^, and frequency distributions of amplicon were analyzed by CRIPResso2 (V2.0.43) ^5^.

**Immunoblotting analysis of ANK2 knockdown**

Double-stranded siRNA specifically targeting human *ANK2* was ordered from GenePharma. The siRNA designs were list below: antisense, 5’-AUAAAUGAUAGUCGUCCGCTT-3’, and sense, 5’- GCGGACGACUAUCAUUUAUTT-3’. The siRNA was transfected into human 293T cells with Lipofectamine 2000 (Invitrogen, 11668-019). Knockdown efficiency was evaluated by immunoblotting analysis at 48 hours after transfection and was used as a control for showing the knockdown of ANK2 isoform.

Human 293T cells with or without siRNA transfection and homogenized brain tissues from wild-type monkey and giant ANK2 knockout monkey (T114) were lysed with RIPA lysis buffer (Beyotime, P0013B) for 20 min in ice. The supernatants were collected after centrifuging at 10000 g at 4 ^o^C for 10 min and subject to immunoblotting blotting analysis. Membrane was incubated with ANK2 antibody (Biolegend, MMS-5246, 1:1000 dilution) followed by incubation with secondary antibody (Thermo Fisher Scientific, 31430, 1:5000 dilution). Images were captured using a Protein SimpleFluorChem system.

**Magnetic resonance imaging (MRI) scanning and quantification**

A detailed description of MRI experiment can be found in our previous studies ^6-9^. Briefly, the MRI data were acquired from a United Imaging UMR 790 3T scanner (Shanghai, China) using a 12-channel knee coil. Before each MRI scanning session, all monkeys were injected intramuscularly with atropine (0.05 mg/kg) and ketamine (5 mg/kg) and sodium pentobarbital (20 mg/kg). Anesthesia was maintained with the lowest possible concentration of sodium pentobarbital under the close supervision of experienced veterinarians. High-resolution T1-weighted anatomical images were acquired using an MPRAGE sequence (TR = 13010 ms; TE = 5600 ms; inversion time = 880 ms; flip angle = 8°; acquisition voxel size = 0.5 × 0.5 × 0.5 mm^3^). For each animal, the first acquired T1-weighted image was selected as a reference and the remaining images from that animal were aligned to it and then averaged to obtain the motion-corrected images using FMRIB’s Linear Image Registration Tool in FSL software (http://www.fmrib.ox.ac.uk/fsl/) ^10^. We applied intensity bias correction for field inhomogeneity to each motion-corrected image using CMTK’s (http://nitrc.org/projects/cmtk/) mrbias tool ^11^. Then, tissue segmentation of T1-weighted images into gray matter (GM), white matter (WM), and cerebrospinal fluid (CSF) was performed using FMRIB’s Automated Segmentation Tool within FSL ^12^. A tissue-probability map of GM was generated to calculate the GM volume for individual brain region. Each GM map was registered to the brain atlas space of macaque monkey by nonlinear Diffeomorphic Anatomical Registration Through Exponentiated Lie Algebra (DARTEL) ^13^ and then modulated to preserve the regional volumetric information of GM tissue within a voxel. This was done by multiplying the intensity value of each voxel in the segmented images by the Jacobian determinants (non-linear components only) that were derived from the spatial registration process. Afterwards, images were smoothed with a 2-mm isotropic Gaussian kernel. Thus, the tissue volumes of GM, WM, and CSF were calculated from the corresponding partial volume maps, and the total brain volume was calculated by summing up these three partial volume maps in the native space. Note that individual brain sizes were corrected for group comparison.

**Measurement of sleep-wake cycle**

The Actical Physical Activity Monitors (Respironics, Pennsylvania, U.S.A.) were used to monitor sleep-wake cycle of free-moving monkeys (*ANK2* KO monkeys and their age- and gender-matched WT control monkeys) for seven consecutive days at three ages ^14^. The Actical monitor uses a single internal omni-directional accelerometer that senses motion in all direction, integrates the amplitude and frequency of detected motion and produces an electrical current varying in magnitude. Therefore, an increased intensity of motion produces an increase in voltage. Actical stores the activity data in the forms of activity counts.

The state of sleep was scored in 1-min epochs to describe the nighttime sleep ^14,15^. When the monkey was observed to exhibit less than three times of body or limb movement within 1 min, the sleep was scored. During relaxed sleep, the animals exhibited no body or limb movements. Meanwhile, the Actical monitors stored the daytime and nighttime activity data, and the ratio of daytime/nighttime activity was also analyzed.

**Behavioral performance**

The social interaction test was used to characterize behavioral performance in monkeys ^15^. This test avoids the use of reward, and does not require extensive training of the animal, making it possible to observe the natural performance of behaviors. The most commonly used test is one-to-one interaction, in which one test monkey (the mutant or its age- and gender-matched WT monkey) is paired with one sociable monkey. The behaviors tested include active and passive social interaction; exploratory and stereotypical behaviors, as well as staying alone and self-grooming. Active social interaction is defined as initiating a play, sharing toys, grooming for others, sitting together (within another monkey’s arms’ reach or in contact) etc., while passive social interaction is defined as receiving aforesaid social interactions from the sociable monkey. Explorative behaviors included tactile exploration of the cage or environments and oral exploration of the cage or environments, which can be used to reflect the monkey’s interests ^16^. The stereotypical behaviors are defined as repetitive and consistent actions with no apparent purposes, including pacing (repetitive, ritualized movement usually involving circling in the cage), digit sucking (sucking on a finger or toe), self-grasping (grabbing or holding onto part of their own body), rocking (a back and forth movement of the upper body with still feet), bouncing (jumping up and down on all four legs), cage shaking (any vigorous shaking of the cage), body spasms (a quick shake of the body), and lip-smacking (pursing the lips together and moving them to produce a smacking sound). The monkey is considered as staying alone when there is no other monkey within its arms’ reach. Self-grooming is defined as cleaning or maintaining the individual’s own body or appearance, which may be indicative of stereotypes. In our experiment, the tested monkeys were recorded once daily (an hour a day) for seven consecutive days. Each video-recording was scored simultaneously by three observers unaware of animal grouping. The observers calculated the frequency and duration of the specific behavior by manual starting and stopping the video under the condition that they all agreed on the classification of the observed behavior. The inter-rater correlation coefficient was found to be > 0.90 through SPSS statistical analysis after a period of training. The duration of each behavior was scored and statistically compared between the KO and WT monkeys.

**Statistical analyses**

Data analysis was conducted using the SPSS version 24.0 software package (SPSS, Chicago, IL, U.S.A.). The normality of the data was analyzed by Kolmogorov-Smirnov tests. We compared the MRI data of each mutant monkey with specie-specific standard brain atlas for rhesus macaque (F99) and cynomolgus macaque (Cyno162) after having segmented each monkey’s brain into 94 sub-regions, instead of using species-matched WT controls as the standard brain atlas was constructed using more individuals ^17,18^. The sleep-wake data were analyzed in separate 2 (groups: KO versus WT) × 3 (age: 9 months, 15 months and 24 months) repeated-measures ANOVAs, with age being the repeated-measure. One-way ANOVA was used to analyze the differences in ASDs-like behaviors between the KO and WT monkeys. The alpha level was set at *p* = 0.05. All *p* values were generated using two-sided tests and all the data were presented as the mean ± SEM (standard error of the mean).

**Supplementary Table S1.** The information for genetically modified monkeys with giant ANK2 targeted-mutations and wild type control monkeys

| **Monkeys** | **Species** | **Gender** | **Delivery mode** | **Rearing pattern** | **Genotype** | **Mutation** |
| --- | --- | --- | --- | --- | --- | --- |
| T87 | Cynomolgus monkey | Male | Natural delivery | Mother-rearing | *ANK2^tm/tm^* | Frameshift |
| T88 | Cynomolgus monkey | Female | Natural delivery | Mother-rearing | *ANK2^tm/tm^* | Frameshift |
| T105 | Rhesus monkey | Male | Natural delivery | Mother-rearing | *ANK2^tm/tm^* | Frameshift |
| T111 | Rhesus monkey | Female | C-section | Nursery/peer-reared | *ANK2^tm/tm^* | Frameshift |
| T113 | Rhesus monkey | Female | C-section | Nursery/peer-reared | *ANK2^tm/tm^* | Missense |
| CWT1 | Cynomolgus monkey | Male | Natural delivery | Mother-reared | *ANK2*^+/+^ | — |
| CWT2 | Cynomolgus monkey | Male | Natural delivery | Mother-reared | *ANK2*^+/+^ | — |
| CWT3 | Cynomolgus monkey | Male | Natural delivery | Mother-reared | *ANK2*^+/+^ | — |
| CWT4 | Cynomolgus monkey | Female | Natural delivery | Mother-reared | *ANK2*^+/+^ | — |
| CWT5 | Cynomolgus monkey | Female | Natural delivery | Mother-reared | *ANK2*^+/+^ | — |
| CWT6 | Cynomolgus monkey | Female | Natural delivery | Mother-reared | *ANK2*^+/+^ | — |
| RWT7 | Rhesus monkey | Male | Natural delivery | Mother-reared | *ANK2*^+/+^ | — |
| RWT8 | Rhesus monkey | Male | Natural delivery | Mother-reared | *ANK2*^+/+^ | — |
| RWT9 | Rhesus monkey | Male | Natural delivery | Mother-reared | *ANK2*^+/+^ | — |
| RWT10 | Rhesus monkey | Female | C-section | Nursery/peer-reared | *ANK2*^+/+^ | — |
| RWT11 | Rhesus monkey | Female | C-section | Nursery/peer-reared | *ANK2*^+/+^ | — |
| T89 | Cynomolgus monkey | Female | C-section | Died at day 1 | *ANK2^tm/tm^* | Frameshift |
| T110 | Rhesus monkey | Male | Natural delivery | Died at day 1 | *ANK2^tm/tm^* | Frameshift |
| T112 | Rhesus monkey | Male | C-section | Died at day 15 | *ANK2^tm/tm^* | Frameshift |
| T114 | Rhesus monkey | Female | C-section | Died at day 17 | *ANK2^tm/tm^* | Frameshift |

Note: Five mutant monkeys (*ANK2^tm/tm^*) were generated in this study, and eleven gender and age-matched wild-type (WT, *ANK2*^+/+^) control monkeys were selected in accordance with the delivery mode of mutant monkeys. If the mutant monkeys were born by natural delivery, the matched WT monkeys were also born by natural delivery. The “CWT” represents the cynomolgus WT monkey, while the “RWT” represents the rhesus WT monkey.

**Supplementary Table S2.** Primer pairs for amplifying sgRNA-targeted regions of the *ANK2* gene

| **Name** | **Sequence (5’-3’)** | **Amplicon** | **Sample** |
| --- | --- | --- | --- |
| ANK2-1F | ACATTACTGGTGGCTCTGAAGA | 723 bp | All |
| ANK2-1R | TCTGCTCAAGACTGTCATCCTC |  |  |
| ANK2-3F | AAGCACCTGCCTATGTCACCTT | 2642 bp | All |
| ANK2-3R | CAATCTACTCGTGGAGATTCTGC |  |  |
| ANK2-HTS1-F^*^ | ACAGTGGAgacagatacaggaactgaatc | 275 bp | T87 |
| ANK2-HTS2-F | GCCAATCGgacagatacaggaactgaatc | 275 bp | T88 |
| ANK2-HTS3-F | ATGTCAGCgacagatacaggaactgaatc | 275 bp | T89 |
| ANK2-HTS4-F | CGATGTgacagatacaggaactgaatc | 273 bp | CWT1 |
| ANK2-HTS5-F | CTTGTAgacagatacaggaactgaatc | 273 bp | CWT2 |
| ANK2-HTS6-F | TGACCAAgacagatacaggaactgaatc | 274 bp | CWT4 |
| ANK2-HTS7-F | CAGATCTgacagatacaggaactgaatc | 274 bp | CWT5 |
| ANK2-HTS8-F | CCGTCCATgacagatacaggaactgaatc | 275 bp | T105 |
| ANK2-HTS9-F | AGTTCCTgacagatacaggaactgaatc | 274 bp | T111 |
| ANK2-HTS9L-F^**^ | AGTTCCTctgtagtgtagcattagcta | 454 bp for WT  271 bp for Mut | T111 |
| ANK2-HTS10-F | GTAGAGCgacagatacaggaactgaatc | 274 bp | T113 |
| ANK2-HTS11-F | GTGGCCTgacagatacaggaactgaatc | 274 bp | T110 |
| ANK2-HTS12-F | CGTACGGgacagatacaggaactgaatc | 274 bp | T112 |
| ANK2-HTS12L-F | CGTACGGgaggtgccacagtcactgag | 569 bp for WT  271 bp for Mut | T112 |
| ANK2-HTS13-F | GAGTGGAGgacagatacaggaactgaatc | 275 bp | T114 |
| ANK2-HTS14-F | AGTCAAgacagatacaggaactgaatc | 273 bp | RWT7 |
| ANK2-HTS16-F | CACGATgacagatacaggaactgaatc | 273 bp | RWT10 |
| ANK2-HTS17-F | TCCCGAgacagatacaggaactgaatc | 273 bp | RWT11 |
| ANK2-HTS-R | ttcatctttggttcaacagg | - | - |

* All ANK2-HTS-(1-17)-F primers are paired with ANK2-HTS-R primer.

** “L” labels forward primer for detecting samples of *ANK2* large deletion.

WT, wild type; Mut, mutant of *ANK2*

**Supplementary Figure S1. Cas9/sgRNA-mediated *ANK2* gene editing detected in embryos of cynomolgus and rhesus monkeys**

(**a**) Genotyping of the *ANK2* alleles in monkey embryos. Upper panel: PCR products of the targeted region of *ANK2* from embryos microinjected with dual sgRNAs. Cynomolgus monkey (*Macaca fascicularis*, Mf) embryos Mf #1-11 were microinjected with 20 ng/µL for each sgRNA and 20 ng/µL Cas9 mRNA, while embryos Mf #14-22 were microinjected with 10 ng/µL for each sgRNA and 20 ng/µL Cas9 mRNA, respectively. Rhesus monkey (*Macaca mulatta*, Mm) embryos Mm #9-13 were microinjected with 20 ng/µL for each sgRNA and 20 ng/µL Cas9 mRNA, respectively. Embryo Mm #12 was PCR amplified using the ANK2-3F/ANK2-3R primer pair that had a PCR product of 2642 bp in wild-type (WT) sample. The ANK2-3F/ANK2-3R primer pair amplified a larger region of *ANK2* compared to the ANK2-1F/ANK2-1R primer pair that yielded a product of 723 bp in WT sample. There was a large fragment deletion in embryo Mm #12. M, DNA ladder; WT, PCR products from respective wild-type cynomolgus and rhesus monkeys. Lower panel: Detection of Cas9/sgRNA-mediated cleavage of *ANK2* by T7EI cleavage assay.

(**b**) Sanger sequencing results of modified *ANK2* alleles. Protospacers are in red text, while PAMs are in green and underlined. Mutations are in blue, and insertions (+), deletions (-) and point mutations (m) are shown on the right of each allele, with rates of clones for TA-sequencing.

(**c**) Frequency of *ANK2-*edited embryos. The counts for genetically modified embryos are labelled on the top of each bar in the histogram, based on the Sanger sequencing results in (**b**).

**Supplementary Figure S2. Knockout of giant ANK2 in monkeys via the Cas9-mediated gene editing**

(**a-b**) Sanger sequencing results (**a**) and deep sequencing results (**b**) of modified *ANK2* alleles in genetically modified monkeys. Genomic DNA isolated from peripheral blood samples was used for sequencing. Protospacers are in red text, PAMs are in green and underlined. Mutations are in blue, and insertions (+), deletions (-) and point mutations (m) are shown on the right of each allele. The rates of clones for TA-sequencing in (**a**) and percentage of high-throughput sequencing reads in (**b**) are presented on the most right column in the respective figure section.

(**c**) The ANK2 protein expression in brain tissues of a wild-type (WT) monkey and giant ANK2 mutant rhesus monkey (T114, KO). Human 293T cells with or without ANK2 knockdown by siRNA were used as a control. The giant ANK2 protein could not be visible on the blot due to its large molecular weight for successful Western blot.

**Supplementary Figure S3. Cas9/sgRNA-mediated *ANK2* gene editing detected in diverse tissues from a dead cynomolgus monkey**

(**a**) Sanger sequencing results of modified *ANK2* alleles in different tissues of a dead cynomolgus monkey (T89). PCR products were cloned into T-vector for sequencing. Protospacers are in red text, with PAM in green and underlined. Mutations are in blue, and insertions (+), deletions (-) and point mutations (m) are shown on the right of each allele, with rates of clones for TA-sequencing.

(**b**) High-throughput sequencing results of modified *ANK2* alleles in brain tissue of monkey T89.

**Supplementary Figure S4. Cas9/sgRNA-mediated *ANK2* gene editing detected in diverse tissues from dead rhesus monkeys**

(**a**) Sanger sequencing results of modified *ANK2* alleles in different tissues of dead rhesus monkeys (T110, T112 and T114). PCR products were cloned into T-vector for sequencing. Protospacers are in red text, with PAM in green and underlined. Mutations are in blue, and insertions (+), deletions (-) and point mutations (m) are shown on the right of each allele, with rates of clones for TA-sequencing.

(**b**) High-throughput sequencing results of modified *ANK2* alleles in brain tissues of T110 and T112 and liver tissue of T114.

**Supplementary Figure S5. MRI imaging showing brain volume loss in giant ANK2 knockout monkeys**

(**a-b**) Example axial slices of structural images of cynomolgus monkeys (**a**: T87 and T88) and rhesus monkeys (**b**: T105 and T111) with giant ANK2 knockout (KO) at the ages of 6 and 12 months. (**c**) Example axial slices of structural images of rhesus monkey T113 and an age-matched wild-type (WT) rhesus monkey. Monkey T113 displayed grossly normal brain structure at all time-points examined. It has ANK2 mutations introduced by gene editing but the mutations may not significantly alter the function of giant ANK2.

**Supplementary Supplementary Figure S6. Giant ANK2 knockout monkeys display reproducible brain volume loss**

(**a**) Regional gray matter volume (GMV) loss for 94 brain regions was averaged across four KO monkeys and mapped on a standard monkey brain surface, in which the hot bar denotes the percentage of GMV loss.

(**b**) The overall distribution of the percentage of regional GMV loss in each KO monkey.

(**c**) Top 20 brain regions with the largest GMV loss are listed for each KO monkey compared to controls in the standard brain atlases. The horizontal axis represents the volume size of each brain region. Regions that exhibited consistent morphological changes in all four mutant monkeys are marked with dark red (light red for three mutant monkeys).

**References**

1 Chen, Y. *et al.* Functional disruption of the dystrophin gene in rhesus monkey using CRISPR/Cas9. *Human molecular genetics* **24**, 3764-3774 (2015).

2 Aronesty, E. Comparison of sequencing utility programs. *The open bioinformatics journal* **7**, 1-8 (2013).

3 Li, H. & Durbin, R. Fast and accurate short read alignment with Burrows–Wheeler transform. *Bioinformatics* **25**, 1754-1760 (2009).

4 Li, H. *et al.* The sequence alignment/map format and SAMtools. *Bioinformatics* **25**, 2078-2079 (2009).

5 Clement, K. *et al.* CRISPResso2 provides accurate and rapid genome editing sequence analysis. *Nat. Biotechnol.* **37**, 224-226 (2019).

6 Wang, Z. *et al.* The relationship of anatomical and functional connectivity to resting-state connectivity in primate somatosensory cortex. *Neuron* **78**, 1116-1126 (2013).

7 Lv, Q. *et al.* Large-Scale Persistent Network Reconfiguration Induced by Ketamine in Anesthetized Monkeys: Relevance to Mood Disorders. *Biol Psychiatry* **79**, 765-775 (2016).

8 Cai, D. C. *et al.* MECP2 Duplication Causes Aberrant GABA Pathways, Circuits and Behaviors in Transgenic Monkeys: Neural Mappings to Patients with Autism. *J Neurosci* **40**, 3799-3814 (2020).

9 Zhan, Y. F. *et al.* Diagnostic Classification for Human Autism and Obsessive-Compulsive Disorder Based on Machine Learning From a Primate Genetic Model. *Am J Psychiat* **178**, 65-76 (2021).

10 Jenkinson, M., Bannister, P., Brady, M. & Smith, S. Improved optimization for the robust and accurate linear registration and motion correction of brain images. *Neuroimage* **17**, 825-841 (2002).

11 Likar, B., Viergever, M. A. & Pernus, F. Retrospective correction of MR intensity inhomogeneity by information minimization. *IEEE Trans Med Imaging* **20**, 1398-1410 (2001).

12 Zhang, Y., Brady, M. & Smith, S. Segmentation of brain MR images through a hidden Markov random field model and the expectation-maximization algorithm. *IEEE Trans Med Imaging* **20**, 45-57 (2001).

13 Ashburner, J. A fast diffeomorphic image registration algorithm. *Neuroimage* **38**, 95-113 (2007).

14 Qin, D. D. *et al.* Potential use of actigraphy to measure sleep in monkeys: comparison with behavioral analysis from videography. *Zoological research* **41**, 437-443 (2020).

15 Chen, Y. *et al.* Modeling Rett syndrome using TALEN-edited MECP2 mutant cynomolgus monkeys. *Cell* **169**, 945-955 (2017).

16 Qin, D. *et al.* The first observation of seasonal affective disorder symptoms in Rhesus macaque. *Behavioural brain research* **292**, 463-469 (2015).

17 Lv, Q. *et al.* Normative analysis of individual brain differences based on a population MRI-based atlas of cynomolgus macaques. *Cerebral Cortex* **31**, 341-355 (2021).

18 Van Essen, D. C. Surface-based approaches to spatial localization and registration in primate cerebral cortex. *Neuroimage* **23**, S97-S107 (2004).
